# Discovering and mapping chromatin states using a tree hidden Markov model

**DOI:** 10.1186/1471-2105-14-S5-S4

**Published:** 2013-04-10

**Authors:** Jacob Biesinger, Yuanfeng Wang, Xiaohui Xie

**Affiliations:** 1 Department of Computer Science, University of California, Irvine, CA, USA; 2 Department of Physics and Astronomy, University of California, Irvine, CA, USA; 3 Institute for Genomics and Bioinformatics, University of California, Irvine, CA, USA

## Abstract

New biological techniques and technological advances in high-throughput sequencing are paving the way for systematic, comprehensive annotation of many genomes, allowing differences between cell types or between disease/normal tissues to be determined with unprecedented breadth. Epigenetic modifications have been shown to exhibit rich diversity between cell types, correlate tightly with cell-type specific gene expression, and changes in epigenetic modifications have been implicated in several diseases. Previous attempts to understand chromatin state have focused on identifying combinations of epigenetic modification, but in cases of multiple cell types, have not considered the lineage of the cells in question.

We present a Bayesian network that uses epigenetic modifications to simultaneously model 1) chromatin mark combinations that give rise to different chromatin states and 2) propensities for transitions between chromatin states through differentiation or disease progression. We apply our model to a recent dataset of histone modifications, covering nine human cell types with nine epigenetic modifications measured for each. Since exact inference in this model is intractable for all the scale of the datasets, we develop several variational approximations and explore their accuracy. Our method exhibits several desirable features including improved accuracy of inferring chromatin states, improved handling of missing data, and linear scaling with dataset size. The source code for our model is available at http:// http://github.com/uci-cbcl/tree-hmm

## Background

Although identical DNA is shared amongst most cells in an organism, a key question in biology relates to how different cell types are formed, maintained, and made to perform vastly different functions. Recent studies have shown that these processes are in part mediated by the post-translational modifications of histone tails, which in turn affect chromatin accessibility and other properties of chromatin structures in a cell-type specific way [1]. There are also interactions between these modifications [2,3], which act combinatorially to exert dynamic control over gene expression and other fundamental cellular processes [4]. Although we do not fully understand the role of epigenetic modifications, their effect in the development of disease and in defining cell type is becoming clearer. For example, epigenetic changes have been shown to be tightly correlated with gene expression [5-7], have been linked to metastasis development in certain types of cancer [8] and are shown to control recombination [9]. Epigenetic inheritance across cells and across individuals has been highlighted in recent research (see [10] for a review) and our understanding of the scope of epigenetic modifications has expanded considerably in recent years.

Chromatin immunoprecipitation coupled with high-throughput sequencing (ChIP-seq) has emerged as a cost-effective method for determining epigenetic modifications. Although initially used as a high-resolution transcription factor binding site discovery mechanism (see [11,12] for review), ChIP-seq has recently been used to target histone tail modifications and is proving to be particularly cost-effective method for epigenomic annotation. Thanks to the ENCODE project [6], hundreds of ChIP-seq datasets are now publicly available and the process of integrating species-specific and cell-type specific binding site information, gene expression, and chromatin state is now underway. These high-throughput datasets provide an unbiased, comprehensive view of the function of different genomic regions.

Several computational approaches have been used to tackle the important problem of genome annotation using these high-throughput datasets. In particular, methods that integrate histone modification data can be segregated into two general approaches: one approach searches near known genomic annotations to identify characteristic marks of particular classes of regions, such as promoters and enhancers, and subsequently uses the learned characteristics to find new instances of the class [13-15]. The other approach learns the characteristic patterns of histone marks *de novo *using unsupervised methods, "rediscovering" and predicting genomic features associated with mark combinations. Methods for identifying these patterns have included clustering [16,17], a dynamic Bayesian network [18], and hidden Markov models (HMM) [6,19,20]. These methods differ mostly in how they model the chromatin mark signal intensity. Some determine a characteristic signal shape while others focus on modeling the mark signal using non-parametric histograms, multivariate normal distributions, or binary presence and mark co-occurrence. Each of these methods focuses on modeling the histone mark combinations; none explicitly incorporate the *lineage *information by which the data are related.

Here, we expand the HMM methodology of Ernst et al. [21] (called ChromHMM), who originally analyzed nine transcription factors (TF) or histone modifications (plus control) performed in nine different human cell types. Their multivariate HMM model concatenated several cell types to form a single chain with the goal of learning a global set of histone mark combinations and left as secondary all comparative analysis between cell types. We generalize the model to more closely reflect biological reality: chromatin remodeling occurs as cells progress through several stages of differentiation. We expect many genomic regions to be correlated across a lineage since cell types diverged from a common progenitor are likely to share the chromatin changes that took place in that progenitor. To capture this reality, we simultaneously model both the genomic localization of histone marks and the chromatin dynamics along a lineage by explicitly aligning each cell type and connecting their internal, hidden nodes vertically in a tree structure. Our model learns both histone modifications' association with chromatin state and state transitions between cell types, capturing epigenetic changes that occur through differentiation or disease progression. Our method effectively pools information across species, and we expect it to show improved accuracy of genome segmentation over the previous HMM approach which does not incorporate cell lineage information.

## Methods

### Tree hidden Markov model

#### Model description and notation

We propose a tree hidden Markov model (TreeHMM) to discover and map chromatin states using the observed chromatin modification data. We begin by introducing some notation. We denote the chromatin modification of type *l *at position *t *of cell type *i *as $x_{t,l}^{i}$, which can take binary values, i.e.$x_{t.l}^{i}\in \left\{0,1\right\}$. Subsequently we denote all the histone marks at position (*i*, *t*) to be $x_{t}^{i}=\left(x_{t,1}^{i},.\;.\;.\;,x_{t,L}^{i}\right)$, which is a vector of length *L *and $X=\left\{x_{t}^{i}:\;i=1,\;.\;.\;.\;,I;t=1,.\;.\;.\;,T\right\}$ to be the collection of all observed data. We further introduce a hidden variable $z_{t}^{i}$ to denote the underlying chromatin state at chromosomal position *t *of cell type *i*. We assume $z_{t}^{i}$'s are discrete taking *K *possible values, i.e., $z_{t}^{i}\in \left\{1,.\;.\;.\;,K\right\}$ for all *t *and *i*. Let $Z=\left\{z_{t}^{i}:\;i=1,\;.\;.\;.\;,I;t=1,.\;.\;.\;,T\right\}$ denote the collection of all hidden chromatin state variables. We assume that these chromatin state variables are the key determinant of the observed chromatin modifications, and that $x_{t}^{i}$'s are independent of each other conditioned on *Z*, i.e., $\mathbb{P}\left(X\;|Z\right)=\prod_{i=1}^{l}\prod_{t=1}^{T}\mathbb{P}\left(x_{t}^{i}|z_{t}^{i}\right)$.

**Figure 1:**
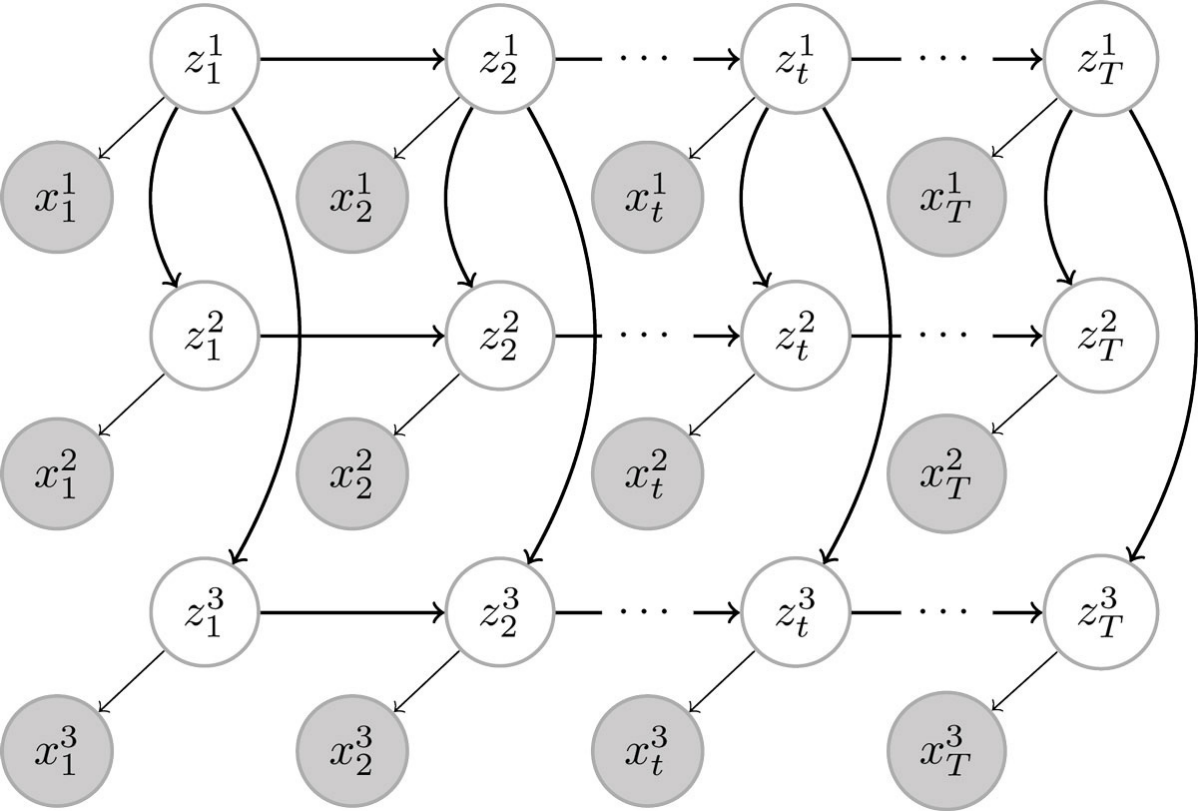
**Example graphical model for a tree-structured HMM with three cell types**. Hidden state variables representing chromatin states (white) are connected horizontally in a chain as well as vertically in a tree structure. Each chain in the graph represents a certain cell type. For example, the top chain represents the root cell type (e.g., ES cells). Observed nodes (grey) represent chromatin modifications and are connected only to the hidden variables.

We assume the *I *cell types are related to each other through a lineage tree T  and use *π*(*i*) to denote the parent node of the cell type *i *within the lineage tree T . The conditional dependencies among the variables are modelled by a Bayesian network as shown in Figure 1 with the chromatin state variables at neighboring positions of each cell type linked as a chain (referred to as horizontal connections) and the state variables of different cell types at the same chromosomal position connected according to the lineage tree T  (referred to as vertical connections). The horizontal connections capture the spatial correlation between chromatin states, i.e., the tendency of histone modifications to spread and cluster spatially across the genome, allowing for example large inactivated regions and short "poised" regions. The lineage relation is modelled by vertical connections between the same locations of different chains, and captures temporal changes in chromatin states during differentiation or disease progression over the cell lineage. Given the conditional dependency specification, the joint distribution of the chromatin state variables can then be written as

(1) $$\mathbb{P}\left(Z\right)\;=\;\overset{I}{\underset{i=1}{\prod }}\overset{T}{\underset{t=1}{\prod }}\mathbb{P}\left(z_{t}^{i}|z_{t-1}^{i},\;z_{t}^{\pi \left(i\right)}\right)$$

where by definition $z_{t-1}^{i}=\emptyset$ if *t *= 1 and $z_{t}^{\pi \left(i\right)}=\emptyset$ if node *i *is the root cell type. As a notation, we also use *π *(*i*, *t*) to denote the parent nodes of node (*i*, *t*) in the model, and use *z*_*π*(*i*, *t*) _to denote the state variables at these parent nodes if they exist.

#### Parameters

The TreeHMM model presented above requires us to specify two sets of conditional distributions. One is the emission probabilities $P\left(x_{t}^{i}|z_{t}^{i}\right)$, that is, the probability of observing chromatin modification vector $x_{t}^{i}$ conditioned on chromatin state $z_{t}^{i}$. For simplicity, we assume different chromatin modification marks are independent of each other conditioned on the chromatin state, and use $e_{l}^{k}=\mathbb{P}\left(x_{t,l}^{i}=1\;|z_{t}^{i}=k\right)$ to denote the probability of observing mark *l *at position *t *of cell type *i *conditioned on the underlying state being *k*.

The second set of conditional probabilities we need to specify are the transition probabilities among chromatin states, that is, $\mathbb{P}\left(z_{t}^{i}|z_{t-1}^{i},\;z_{t}^{\pi \left(i\right)}\right)$. When *t *> 1 and *π*(*i*) is not empty, we will use a *K *× *K *× *K *matrix to specify $\mathbb{P}\left(z_{t}^{i}|z_{t-1}^{i},\;z_{t}^{\pi \left(i\right)}\right)$. However, when one of the conditioned variable is non-existent, we use *K *× *K *matrix to specify the transition probability. More specifically, the state transition probabilities are

$$\begin{array}{cc} \theta_{bc}^{a}=\mathbb{P}\left(z_{t}^{i}=a|z_{t}^{\pi \left(i\right)}=b,\;z_{t-1}^{i}=c\right) & t\neq 1,\;i\;\text{is}\;\text{not}\;\text{root}\\ \alpha_{b}^{a}=\mathbb{P}\left(z_{t}^{i}=a|z_{t-1}^{i}=b\right) & t\neq 1,\;i\;\text{is}\;\text{root}\\ \beta_{b}^{a}=\mathbb{P}\left(z_{t}^{i}=a|z_{t}^{\pi \left(i\right)}=b\right) & t=1,\;i\;\text{is}\;\text{not}\;\text{root}\\ \gamma^{a}=\mathbb{P}\left(z_{t}^{i}=a\right) & t=1,\;i\;\text{is}\;\text{root}. \end{array}$$

We will also use $\Theta =\left\{\theta_{bc}^{a},\;\alpha_{b}^{a},\;\beta_{b}^{a},\;\gamma^{a},\;e_{l}^{a}|\left(a,\;b,\;c\right)\in 1,\;\ldots ,\;K;l\in 1,\;\ldots ,\;L\right\}$ to denote the collection of all parameters associated with the model.

### Inference and parameter learning

Given the TreeHMM model described above and the set of observed chromatin modification data *X*, our goal is to: 1) estimate the parameters of the model, and 2) infer the underlying hidden state at each chromosomal location of each cell type. For parameter learning, we will use the maximum likelihood method, that is, we seek to find the optimal parameter set Θ* that maximizes the log likelihood function

(2) $$\text{log}L\left(\Theta ;X\right)=\text{log}\mathbb{P}\left(X;\Theta \right)=\text{log}\underset{z}{\sum }\mathbb{P}\left(Z;\Theta \right)\mathbb{P}\left(X|Z;\Theta \right)$$

Note that in the above notation, we put Θ into the distributions to emphasize the dependency of the distributions on the parameters. However, we will also the simplified notation ℙ(Z|X) or ℙ(X) when the context is clear. After finding the optimal parameters, we infer the underlying chromatin states using posterior inference, to calculating the posterior probability of each chromatin state conditioned on the observed data, $\mathbb{P}\left(z_{t}^{i}\;|\;X;\Theta \right)$.

We explore various inference methods for the TreeHMM model, including exact methods and approximate methods. For exact inference, we provide two implementations: first, we generate a lattice for the Graphical Models Toolkit (GMTK) [22], which provides an efficient framework for exact inference and learning using the junction tree algorithm [23]. We also provide a custom library which implements a "cliqued" method in which each slice *t *of the model has all its nodes in that slice treated as if they were part of a single "cliqued" node that has *K^I ^*states. In this cliqued node representation, we can apply standard HMM methodology to do inference and learning. The state space of the cliqued inference method grows exponentially with *I*, but we found it to be faster than the GMTK implementation for small trees. Both implementations gave the same results in our testing.

Since the TreeHMM model contains undirected cycles, exact inference methods such as junction tree and the "cliqued" method quickly become intractable in computational time and memory consumption when the number of nodes *I *or the number of inferred states *K *increases. Therefore, we introduce several approximate inference methods to solve the inference and learning problem presented above. We focus on variational methods since they are usually computationally efficient and scale well with size of the dataset [24]. The overall strategy of variational methods is to find an easier-to-handle surrogate distribution of the states ℚ(Z) that can be used to approximate the true posterior distribution ℙ(Z|X) This is done through the venue of the free energy function

(3) $$F=-\underset{Z}{\sum }\mathbb{Q}\left(Z\right)\text{log}\frac{\mathbb{P}\left(X,Z;\Theta \right)}{\mathbb{Q}\left(Z\right)}=E_{\mathbb{Q}}\left[\text{log}\mathbb{Q}\left(Z\right)\right]-E_{\mathbb{Q}}\left[\text{log}\mathbb{P}\left(X,\;Z;\Theta \right)\right]$$

By Jensen's inequality, *F *is always lower bounded by the negative log likelihood function, i.e.F≥-logL(Θ;X), with equality holding if and only if ℚ(Z)=ℙ(Z|X). The goal of the variational inference is to find a ℚ  distribution (usually under some approximate form) that minimizes the free energy function. We will consider three different forms of surrogate distributions and briefly describe variational inference for each of them. Details of the derivations are given in Additional file 1, section 1.3.

#### Mean field (MF) variational inference

In the mean field variational method, we consider the surrogate distribution to be the product of the marginal distributions of each individual state variable

(4) $$\mathbb{Q}\left(Z\right)=\overset{I}{\underset{i=1}{\prod }}\overset{T}{\underset{t=1}{\prod }}q\left(z_{t}^{i}\right)$$

where $q\left(z_{t}^{i}\right)$ represents the marginal distribution of $z_{t}^{i}$. For notational simplicity, we also use *q_it _*as an abbreviation of $q\left(z_{t}^{i}\right)$. In this case, the free energy becomes

(5) $$F=\overset{I}{\underset{i=1}{\sum }}\overset{T}{\underset{t=1}{\sum }}E\left[\text{log}q\left(z_{t}^{i}\right)-\text{log}\mathbb{P}\left(x_{t}^{i}\;|z_{t}^{i}\right)\right]-E\left[\text{log}\mathbb{P}\left(z_{t}^{i}\;|z_{\pi \left(i,t\right)}\right)\right]$$

where the expectation is with respect to ℚ , as will always be the case in the remainder of this paper.

To find the optimal ℚ  that minimizes the free energy, we use a coordinate descent method - alternatively updating each component *q_it _*while keeping all other components fixed. To update *q_it _*we collect the terms in *F *that involve *q_it_*,

$$F_{it}=E\left[\text{log}q\left(z_{t}^{i}\right)-\text{log}\mathbb{P}\left(x_{t}^{i}|z_{t}^{i}\right)\right]-E\left[\text{log}\mathbb{P}\left(z_{t}^{i}|z_{\pi \left(i,t\right)}\right)\right]-\underset{\left\{\left(j,s\right):\left(i,t\right)\;\in \pi \left(j,s\right)\right\}}{\sum }E\left[\text{log}\mathbb{P}\left(z_{s}^{j}|z_{\pi \left(j,s\right)}\right)\right].$$

The last term involves nodes that are children of (*i*, *t*). The update formula for *q_it _*is thus given by $q\left(z_{t}^{i}\right)\sim \text{exp}\left\{\phi \left(z_{t}^{i}\right)\right\}$, up to a normalizing constant, where

$$\phi \left(z_{t}^{i}\right)=\text{log}\mathbb{P}\left(x_{t}^{i}|z_{t}^{i}\right)+E_{q_{\pi \left(i,\;t\right)}}\left[\text{log}\mathbb{P}\left(z_{t}^{i}|z_{\pi \left(i,t\right)}\right)\right]+\underset{\left\{\left(j,s\right):\left(i,t\right)\in \pi \left(j,s\right)\right\}}{\sum }E_{q_{j\;\text{s}},q_{\pi \left(j,\;\text{s}\right)\backslash \left(i,\;t\right)}}\left[\text{log}\mathbb{P}\left(z_{s}^{j}|z_{\pi \left(j,s\right)}\right)\right].$$

The (*j*, *s*) nodes in the last term are all children of node (*i*, *t*), but the expectation involves all the parents of (*j*, *s*) except (*i*, *t*).

#### Structured mean field(SMF) variational inference

In the structured mean field variational method, we consider the surrogate distribution to be the product of the marginal distributions of disjoint sets of state variables. Let $z_{i}=\left\{z_{t}^{i}\;:\;t=1,\;\ldots ,\;T\right\}$ denote the set of all state variables within cell type *i*, corresponding to the state variables within each horizontal chain of the TreeHMM model. We consider the ℚ  to be of the following form

(6) $$\mathbb{Q}\left(Z\right)=\overset{I}{\underset{i=1}{\prod }}q_{i}\left(z_{i}\right),$$

written as the product of marginal distributions of **z***_i _*variables. In this case, the free energy becomes

(7) $$F=\overset{I}{\underset{i=1}{\sum }}\left[E\left[\text{log}q_{i}\left(z_{i}\right)\right]-\overset{T}{\underset{t=1}{\sum }}\left(E\left[\text{log}\mathbb{P}\left(z_{t}^{i}|z_{\pi \left(i,t\right)}\right)\right]+E\left[\text{log}\mathbb{P}\left(x_{t}^{i}|z_{t}^{i}\right)\right]\right)\right]\;.$$

To find the optimal distribution ℚ  that minimizes the free energy, we again alternatively optimize each marginal distribution component while keeping others fixed. To update *q_i_*(**z***_i_*), we collect the terms in *F *that involve *q_i_*(**z***_i_*),

(8) $$F_{i}=E_{q_{i}}\left[\text{log}q_{i}\left(z_{i}\right)-\overset{T}{\underset{t=1}{\sum }}\left(\text{log}f_{it}\left(z_{t}^{i},\;z_{t-1}^{i}\right)+\text{log}\mathbb{P}\left(x_{t}^{i}|z_{t}^{i}\right)\right)\right],$$

where we have defined $f_{it}\left(z_{t}^{i},\;z_{t-1}^{i}\right)=\text{exp}\left\{E_{q_{\pi \left(i\right)}}\left[\text{log}\mathbb{P}\left(z_{t}^{i}|z_{\pi \left(t,i\right)}\right)\right]+\sum_{j:i=\pi \left(j\right)}E_{q_{j}}\left[\text{log}\mathbb{P}\left(z_{t}^{j}|z_{\pi \left(j,t\right)}\right)\right]\right\}$. Since *f_it _*only involves expectations with respect to the distributions other than *q_i_*, it is a fixed function of $z_{t}^{i}$ and $z_{t-1}^{i}$ during the update of the *q_i_*(**z***_i_*). If the *f_it _*functions can be normalized to be conditional probability distributions, then Equation (8) shares the exact form of the free energy of a hidden Markov model with transmission probabilities specified by $f_{it}$ and emission probabilities specified by $\mathbb{P}\left(x_{t}^{i}|z_{t}^{i}\right)$. As such, the optimal *q_i _*minimizing the free energy is the same as the posterior probabilities of the states in the hidden Markov model, which can be efficiently calculated using the forward-backward algorithm [25]. The details of how to normalize the *f_it _*functions to be proper transition probabilities are shown in Additional file 1, section 1.3.

#### Loopy belief propagation (LBP)

The third inference method we used is loopy belief propagation. Belief propagation is a message passing algorithm commonly used in probabilistic graphical models. The algorithm is exact for tree and poly-tree structured graphs. For general graphs that contain cycles or loops, it is an approximate algorithm also called loopy belief propagation. In this case, the algorithm is not guaranteed to converge nor is the approximate free-energy a bound of the log-likelihood. Nevertheless, it has shown empirical success in some cases [26]. Loopy belief propagation can be also viewed as a variational method with the ℚ  distribution taking the Bethe approximation form upon convergence [27]. Here we use Pearl's belief propagation algorithm which is directly applicable to the Bayesian network representation. We refer readers to [28] for the details of the algorithm.

#### Parameter learning

Above we have introduced different inference methods. To do parameter learning, we use a variant of the expectation-maximization (EM) algorithm called variational EM algorithm. Like the EM algorithm, the variational EM algorithm iterates between two steps: an expectation and a maximization step. The expectation step (E-step) is performed by the inference methods, during which we calculate ℚ(Z) in the approximate forms outlined above with fixed parameter values. In the maximization step (M-step), we seek parameter values that minimize *F *(or maximize -*F*) under ℚ(Z).

Consider the free energy *F *as a function of Θ, the variational maximization step seeks the parameters that minimize *F *given the current hidden variable distribution ℚ(Z), i.e.

$$\hat{\Theta }=\text{arg}\underset{\Theta }{\text{min}}F\left(\Theta ,\;\mathbb{Q}\left(Z\right)\right).$$

The above optimization can be solved explicitly. As a result, the state transition parameters are calculated as $\theta_{bc}^{a}\propto \sum_{i>1}\sum_{t>1}\mathbb{Q}\left(z_{t}^{i}=a,\;z_{t}^{\pi \left(i\right)}=b,\;z_{t-1}^{i}=c\right)$, $\alpha_{b}^{a}\propto \sum_{t>1}\mathbb{Q}\left(z_{t}^{1}=a,\;z_{t-1}^{1}=b\right)$, $\beta_{b}^{a}\propto \sum_{i>1}\mathbb{Q}\left(z_{1}^{i}=a,z_{1}^{\pi \left(i\right)}=b\right)$, $\gamma^{a}\propto \mathbb{Q}\left(z_{1}^{1}=a\right)$ up to a normalization constant, where ℚ(⋅) denotes the marginal distribution of the variables inside the brackets. The emission parameters are given by $e_{l}^{a}=\frac{\Sigma_{i,t}\mathbb{Q}\left(z_{t}^{i}\;=\;a\right)I\left(x_{t,l}^{i}\;=\;1\right)}{\Sigma_{i,t}\mathbb{Q}\left(z_{t}^{i}=a\right)}$ where *I*(·) is the indicator function. The variational EM algorithm for the SMF case is outlined in Algorithm 1. Notationally, we have considered the entire genome as a single chunk. In practice, we break up the genome into many smaller chunks to allow more efficient, parallel execution and to reduce memory consumption, at the cost of computational artifacts at chunk borders.

### Data processing

As a preprocessing step, we create a histogram of mapped reads by dividing the genome into 200 bp non-overlapping bins and counting the number of mapped reads whose middle base fell into each bin. All replicates, if any, were added to the histogram and the histogram was then binarized using a threshold corresponding to a Poisson *p*-value of 10^-4^, similar to [21]. We further segmented the genome into regions with and without chromatin marks by applying a smoothing filter to the raw count data, retaining regions that contained mapped reads. Further data processing details can be found in Additional file 1, section 1.1, and all preprocessing methods are available as part of the released source code.

Our model's preprocessing and parameterization are very similar to the multivariate HMM methodology of [21], however Ernst's implementation suffered from a very slow runtime on our processed data, which contains many regions to facilitate parallel inference. We re-implemented the method as described [20] and use this implementation for comparison in later sections. The implementation is available in the released source code.

### Results

We used the same human ENCODE dataset reported in [21] which contains ChIP-seq profiles for nine human cell types including human embryonic stem cells (H1 ES), erythrocytic leukaemia cells (K562), B-lymphoblastoid cells (GM12878), hepatocellular carcinoma cells (HepG2), umbilical vein endothelial cells (HUVEC), skeletal muscle myoblasts (HSMM), normal lung fibroblasts (NHLF), normal epidermal ker-atinocytes (NHEK), and mammary epithelial cells (HMEC). For each cell type, ten different markers are used including eight histone modifications (H3K27me3, H3K36me3, H4K20me1, H3K4me1, H3K4me2, H3K4me3, H3K27ac, and H3K9ac), one transcription factor closely related to chromatin dynamics (CTCF), and a control data set (whole cell extract). Altogether, the dataset contains 90 ChIP-seq profiles, which were downloaded from the ENCODE website [29].

Since the cell types in the ENCODE data represent very diverse, distinct cell types, we used a simple lineage tree structure with the H1 ES cell type forming the tree root and all other cell types connecting to it directly as leaves. ES cells exhibit unique epigentic biology [30], however hierarchical clustering of the observed marks reveals that each mark exhibits substantial correlation between all cell types (see Supplemental Figure 2 (Additional file 1)). Further, TreeHMM can incorporate information from marks that are only available in certain cell types and can be adapted to more interesting tree structures by including additional latent cell types. Although the current choice of tree structure may be an oversimplification of the underlying biology, we are mostly focusing on the *methodology *for approximate inference in TreeHMM; we explored the performance on artificial data with more interesting tree structures in Additional file 1, section 1.5. Finally, we note that while exact inference methods scale exponentially in the tree width, the approximate inference methods developed here scale linearly with *I*, allowing deeper lineages and more complex tree structures to be examined eventually.

**Figure 2:**
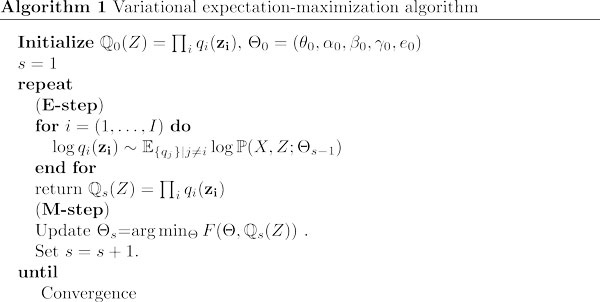
**The variational E-M algorithm**.

#### Comparing approximate inference methods

**Figure 3:**
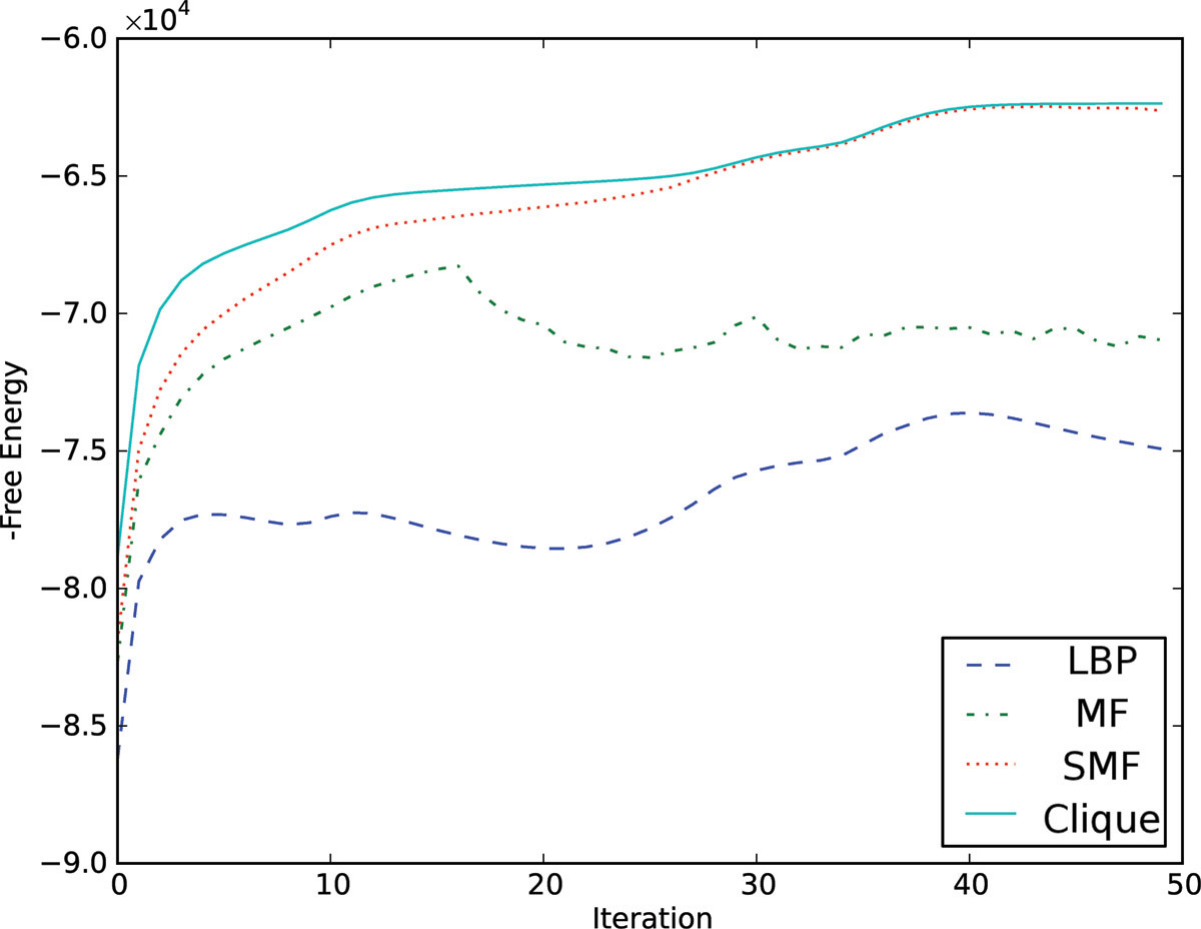
**Free energy for approximate inference methods**. Free energy for different inference methods are compared, with parameters learned using exact inference. The test dataset is restricted to a 2 MB region of chromosome 22 with only three cell types and *K *= 5. The approximate methods use the parameters (learned by the exact method) and only perform inference steps. Note that for the exact algorithm (clique), the free energy equals the negative log-likelihood.

To determine the accuracy of our approximate inference methods, we apply the TreeHMM model to the human ENCODE dataset described above using the following scheme: Exact inference and learning are used to define a set of parameters at each iteration. Each of the approximate inference methods performs inference on the parameters' values to get the free energy. We apply this procedure on a randomly selected 2 MB region with 3 cell types (H1 ES, K562, GM12878) using *K *= 5. Figure 3 shows the log likelihood of the exact inference and the corresponding free energy of different inference methods during exact EM iterations. We observe that the SMF approximation gives the highest negative free energy in this test dataset. The closeness between SMF free energy and the exact log likelihood indicates that the SMF method captures the majority of correlation between variables. Notably, the free energy curves of MF approximation and LBP fluctuate widely as the parameters are refined by the exact algorithm, indicating inconsistency in the free energy landscapes of these approximations and the true one. We also experiment with parameter recovery in several artificial datasets with different tree structures (Additional file 1, section 1.5), and observe that SMF typically outperforms the other approximate methods. As SMF seems to be the most accurate approximation in both the artificial and real data cases, we proceed with the SMF approximation in the following real data genomic segmentation and prediction problems.

#### TreeHMM on the complete genome using the SMF approximation

**Figure 4:**
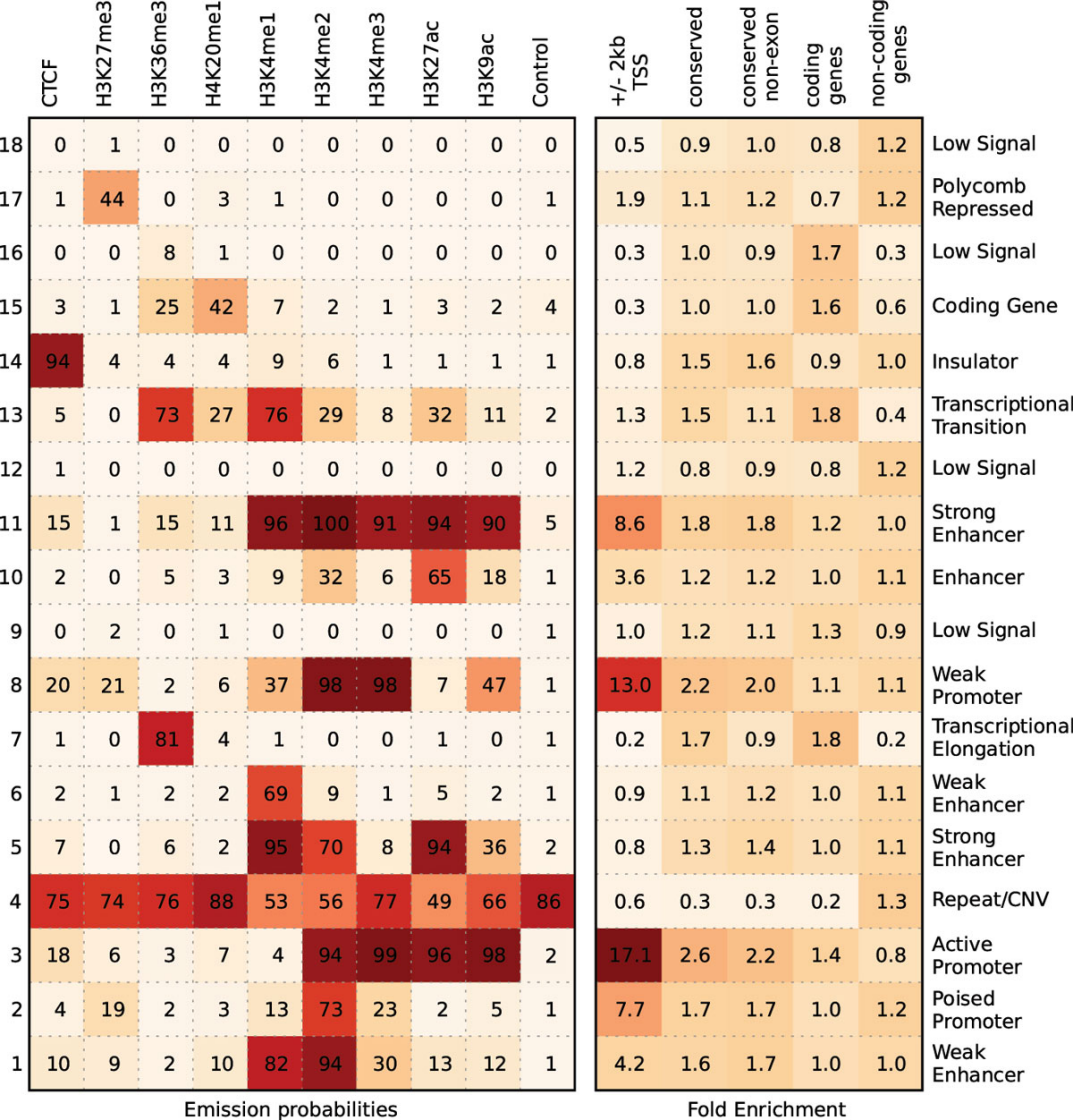
**Learned chromatin states with the associated chromatin modification and enrichment in distinct genomic regions - human data**. Left panel: the probability of observing each histone mark in each of 18 hidden states is summarized. Right panel: fold enrichment of hidden states in various genomic regions reveals strong positional preferences of learned chromatin states.

We next apply the TreeHMM model's SMF approximation to the complete genomic histone data. We use the Bayes Information Criterion, a complexity-penalized likelihood, to determine the optimal number of states *K *= 18 (see Additional file 1, section 1.6). After running several random initializations of the EM algorithm to convergence, we report the one with highest final likelihood. Figure 4 shows the learned states' characteristic chromatin modification co-occurrence patterns (the emission matrix *e*) and their enrichment in different genomic regions. Although states are learned *de novo *based only on the chromatin markers, many marker co-occurrences correspond to previous biological observations (e.g. H3K4 di- and tri-methylation in promoter regions and H3K4 mono- and di-methylation in enhancer regions [31]). We have annotated the likely function of each state (Figure 4) based on its genomic localization and concordance with previously reported findings [21]. The states show distinct enrichment patterns in different genomic locations. Several of the states (3, 8, and 11) are strongly enriched (8-17 fold) in the **±**2 kb TSS region. Other states (7, 13, and 15) are enriched (2-3 fold) in coding genes. The coverage of each chromatin state region also varies widely, as shown in Supplementary Table 2 (Additional file 1). The promoter and enhancer states cover a relatively small portion of the genome, e.g. ~ 1.1% for both active promoter and strong enhancer regions while low signal regions combine for around 75% of the genome. The state distribution also shows some cell-type specific properties, e.g., enhancer states 5, 10 and 11 are largely depleted in H1 ES cells, while other enhancer states are not (one being 2 fold enriched), indicating different functional roles of the learned enhancer states.

To explore the cell-type specificity of our learned states, we performed *K*-means clustering regions assigned to each state in any cell type. We show three of the states in Supplementary Figure 7 (Additional file 1), including the insulator regions (state 14), strong enhancer regions (state 5) and active promoter regions (state 3). We can see that the distribution of different states across cell types differs drastically. Almost half of all insulator sites (state 14) are shared amongst all nine cell types or are only missing in one or two cell types. Many of the remainder are specific to a single cell type. Likewise, some active promoter regions (state 3) are shared amongst all or most cell types, but many more of the promoter regions are cell-type specific. Finally, the strong enhancer regions (state 5) are almost entirely cell-type specific. These overall patterns of cell-type specificity are captured by the learned transition matrices *α *and *θ*, which are shown in Supplemental Figures 3 and 4 (Additional file 1).

Several states are dominated by their vertical component in the *θ *transition matrix, including the states localizing to TSS's (states 2, 3, 8, 10, and 11), copy number variant/repeat regions (state 4), and the insulator state marked by CTCF (state 14). Other states have weak vertical components: consistent with the cell-type specificity of enhancers and chromatin remodeling, three of the enhancer regions (states 1, 5 and 6) and the polycomb repressed regions (state 17) show little to no vertical correlation. In particular, enhancer state 1 does not show the vertical correlation that might be expected given its propensity for TSS regions (4.24 fold enrichment).

#### Comparison with ChromHMM

We compare our result with ChromHMM - a similar method based on hidden Markov model described in [21] that does not utilize lineage information. We ran the HMM on the same histone data, treating each cell type's segment as a separate chain with inference performed in parallel but with tied parameters. We set number of states to be the same as in the TreeHMM result for consistency.

The learned emission probability matrix from ChromHMM together with the confusion matrix between the assigned states of the two results is shown in Supplemental Figure 5 (right panel, Additional file 1). Comparing the emission matrix from two methods (Figure 4 and Supplemental Figure 5 (left panel, Additional file 1)), we observe similar co-occurrence patterns of markers. But as revealed by the confusion matrix, there is a substantial set of regions that are assigned different states due to the lineage constraint introduced in our model. For example, the weak promoter state (state 8) overlaps with ChromHMM's inactive promoter and enhancer states (2 and 8). Also ChromHMM exhibits two repetitive states (similar to [21]) while there is only one such state in the TreeHMM result. To assess the accuracy of our methods, we tested our predicted states' overlap with several human ES-cell-specific ChIP-seq datasets.

We use a recent series of ChIP-seq datasets of transcription factor binding in H1-ES cells [32] including Taf1, p300, Nanog, Klf4, Oct4, and Sox2. Among those, Taf1 is part of the machinery that recruits Polymerase II to the transcription start site and we expect its enrichment in promoter regions. p300 is a transcription factor (TF) that interacts with many other TFs in enhancer regions and we expect its presence in predicted enhancer regions. The other TFs in this dataset are important in maintaining stem-cell state, but a preference for promoter vs. enhancer has not been established. We investigated the overlap of ChIP-seq peaks in these datasets with our predicted states. For each method, we pooled the "enhancer" states (states 1, 5, 6, 10 and 11 in both methods) and report the fraction of sites overlapping called peaks for each transcription factor in Table 1. Similar results are reported for "promoter" regions (states 2, 3 and 8 in both methods).

**Table 1:** H1-ES ChIP-seq enrichment in predicted promoter and enhancer regions.

Promoters				
**Factor**	**TreeHMM**		**ChromHMM**	
	**All**	**Unique**	**All**	**Unique**

Taf1	32,069 (41.6x)	1,489 (15.2x)	35,082 (26.0x)	4,502 (6.7x)
Oct4	4,980 (23.8x)	231 (8.7x)	6,932 (19x)	2,183 (12x)
Klf4	2,622 (18.1x)	105 (5.7x)	3,819 (15.1x)	1,302 (10.3x)
p300	141 (1.0x)	16 (0.9x)	1,597 (6.4x)	1,472 (11.8x)
Nanog	1,556 (1.5x)	227 (1.7x)	8,650 (4.7x)	7,321 (7.7x)
Sox2	412 (1.6x)	63 (2.0x)	2,509 (5.7x)	2,160 (9.8x)

**Enhancers**				

Factor	TreeHMM		ChromHMM	
	All	Unique	All	Unique

Taf1	8,095 (2.5x)	4,293 (4.4x)	5,611 (2.2x)	1,809 (5.3x)
Oct4	3,914 (4.5x)	2,060 (7.8x)	2,274 (3.3x)	420 (4.5x)
Klf4	2,143 (3.6x)	1,294 (7.1x)	1,003 (2.1x)	154 (2.4x)
p300	7,253 (12.2x)	1,517 (8.4x)	5,861 (12.2x)	125 (2.0x)
Nanog	39,829 (9.1x)	7,941 (6.0x)	33,561 (9.6x)	1,673 (3.5x)
Sox2	9,786 (9.4x)	2,185 (6.9x)	7,952 (9.5x)	351 (3.1x)

As shown in Table 1, Taf1 shows strong enrichment in the promoter regions annotated by both ChromHMM and TreeHMM methods (26 and 41.6 fold enrichment over background, respectively). Although the two methods identify a similar number of active promoters (136,702 for TreeHMM vs. 239,792 by ChromHMM), a larger fraction of TreeHMM's predicted promoter overlaps with Taf1 binding sites than ChromHMM (32,069 or 23.5% of sites predicted by TreeHMM vs. 35,082 or 18.5% of sites predicted by ChromHMM). The enhancer regions predicted by the two methods with similar fold enrichment (12.2 and 12.3 fold) in p300 ChIP-seq binding peaks, but 24% more sites are correctly predicted by TreeHMM (7,253 vs. 5,861). An interesting observation is that Oct4 and Klf4 both show preference for promoter regions over enhancer regions and in these cases, ChromHMM captures more of the ChIP-Seq binding sites but at the cost of calling many more total sites (23.8 vs. 19 fold enrichment of Oct4; 18.1 vs. 15.1 fold enrichment of Klf4). Distinctly, Nanog and Sox2 show a strong preference for enhancer regions. For these predictions, more ChIP binding sites (19% more for Nanog, 23% more for Sox2) are captured by TreeHMM at similar enrichment levels. These results indicate TreeHMM's lower false negative rate for enhancer regions and lower false positive rate for promoter regions.

We also investigated the recovery of active transcription start sites. We compared the predicted promoter regions (states 2, 3, and 8) with the ENCODE Capped Analysis Gene Expression (CAGE) data for H1-ES and K562 cells. Supplemental Figure 3 (Additional file 1) shows TreeHMM's improved precision (5-9% better) and similar recall (2% worse to 0.5% better) in predicting active TSS regions.

## Discussion

We have here presented a tree hidden Markov model for identifying chromatin state based on measurements from multiple cell types in a principled way. The major improvement over the previous HMM approach is the incorporation of cell lineage explicitly in the model. While previous methods have focused only on the marks present at a particular region in a particular cell type, we pool information across the same genomic location at different cell types. This allows increased discernment in regions of uncertainty. Although model learning in our proposed model is intractable except in the smallest cases, we developed several approximate methods and demonstrated their accuracy using the ENCODE histone modification data for nine different cell types. Interestingly, we found strong correlations along cell lineages and show that in many cases the information gained from lineage correlations increases state inference accuracy. Inherent to our method is the discovery of states that are more likely to change during differentiation or disease progression. This information allows more accurate prediction and allows accurate delineation between housekeeping genes present in all cell types and genes regulated in a lineage-specific fashion.

In this work, we have focused on developing approximate methods for doing inference and learning in the general framework. Our implementation is general and can deal with missing marks and missing species (discussed in Additional file 1, section 1.4). With the capabilities of the model, there can be many further improvements including incorporating more cell types with incomplete measurements, modifying the lineage tree to include hidden nodes, and incorporating heterogenous data beyond histone marks. By pooling information from similar cell types and learning combinations of marks, it should be possible to infer cell state without a full spectrum of histone modifications measurements. We plan on exploring the rapidly increasingly heterogenous datasets to gain further insight into role of chromatin modifications in determining epigenetic states and their relationship with disease phenotype. Another possible application of the framework is to look into cross-species correlation of histone modification [33] to gain insight into inter-species conservation or divergence of epigenetic mechanisms.

## Conclusions

Understanding epigenetic factors' associations with cell state is a primary step towards proper context for biological systems. Histone modifications play an essential role in regulating and maintaining gene expression and determining cell state. We have developed a novel graphical model for determining chromatin state from epigenetic modifications. Our method explicitly models transitions between cell types during differentiation or disease progression by considering cell lineage relationship. Although performing exact inference in our model is intractable, we develop highly accurate approximate inference methods that scale well with dataset size. By utilizing information from several cell types, our method can infer epigenetic state more accurately and has the ability to incorporate tendency of transitions between cell states in a more principled way. These cross-cell type correlations may be especially useful in datasets where the complete battery of experiments have not been performed in all cell types.

## List of abbreviations used

MF: Mean Field; SMF: Structured Mean Field; LBP: loopy belief propagation; BIC: Bayes Information Criterion; CAGE: Capped Analysis Gene Expression

## Competing interests

The authors declare that they have no competing interests.

## Authors' contributions

JB and YW implemented the methodology. All authors worked on, read and approved the final manuscript.

## Supplementary Material

Additional file 1 **Supplemental material**. Additional details on data processing, model derivation, model parametrization, and training results on the ENCODE and synthetic datasets are available in Additional file 1. Click here for file

## Declarations

Publication of this article was supported by National Institutes of Health grant T15LM07443.

This article has been published as part of *BMC Bioinformatics *Volume 14 Supplement 5, 2013: Proceedings of the Third Annual RECOMB Satellite Workshop on Massively Parallel Sequencing (RECOMB-seq 2013). The full contents of the supplement are available online at http://www.biomedcentral.com/bmcbioinformatics/supplements/14/S5.
