# Supplementary material for: Discovering and mapping chromatin states using a tree hidden Markov model
Source: BMC Bioinformatics. 2013 Apr 10;14(Suppl 5):S4. doi: 10.1186/1471-2105-14-S5-S4 (PMC3622631; doi:10.1186/1471-2105-14-S5-S4)
Supplement: Additional file 1 — Supplemental material. Additional details on data processing, model derivation, model parametrization, and training results on the ENCODE and synthetic datasets are available in Additional file 1. [file 1471-2105-14-S5-S4-S1.PDF]

Additional file 1: supplemental material for discovering and mapping chromatin states using a tree hidden Markov model

Jacob Biesinger[1,3,†] , Yuanfeng Wang[2,†] and Xiaohui Xie*[1,3]

[1]Department of Computer Science, University of California, Irvine
[2]Department of Physics and Astronomy, University of California, Irvine
[3]Institute for Genomics and Bioinformatics, University of California, Irvine
† Contributed equally to this work

Email: Jacob Biesinger - jake.biesinger@uci.edu; Yuanfen Wang - yuanfenw@uci.edu; Xiaohui Xie*- xhx@ics.uci.edu;
*Corresponding author

Supplemental material

1 Supplementary Text

1.1 Data processing for ENCODE dataset

We preprocessed the datasets by dividing the genome into 200-bp non-overlapping bins and then binarized the reads within each bin, similar to [1]. For binarization, we assign value 1 if the total number of reads located within the bin is above the threshold corresponding to a p-value of 10^{-4} under a Poisson model, where the Poisson rate λ is the number of reads in all replicates of an experiment divided by the length of the genome.

To reduce computational cost, we segmented the genome into regions with and without chromatin marks and only use the regions with sufficient reads present. To do this, binned read counts across all species and all marks were summed together into a single track and convolved using a 1-D Gaussian kernel acting over $\sigma = 40$kb. Only regions with at least 0.5 smoothed reads across at least 5kb were retained as having sufficient signal to include in training. In total, these segments covered 54.8% of the genome and inference proceeded on each segment in parallel.

1.2 Model description and parametrization

We use a Bayesian network to model the chromatin states across the genome of I different cell types. The genomic location is divided into total of T fixed-size bins. Each bin is associated with a hidden variable (node) representing the underlying chromatin state and several observed nodes denote the measured chromatin markers. Nodes within one cell type are connected horizontally while cell lineage is modeled by connecting nodes at the same horizontal location in a lineage tree. The resulting model is a TreeHMM model as shown in Fig. 1 of the main article. Each node can be indexed by (i, t) with $i \in \{1, 2, \ldots, I\}$ indicating the cell type and $t \in \{1, 2, \ldots, T\}$ indicating the location. We denote the hidden variables as z_t^i and the observed

variables as x_t^i. The possible values of the hidden and observed variables can take are

$$z_t^i \in \{1, \dots, K\}, x_t^i \in \{0,1\}^L$$

where K is the number of chromatin states and L is the number of different epigenetic markers. Additionally, we will define the following functions for notational simplicity:

$$\begin{aligned}\text{pa}(i,t) &= \{(j,s)| \text{ node } (j,s) \text{ is a parent of } (i,t)\} \\ &= \begin{cases} \emptyset & \text{if } i \text{ is the root and } t = 1 \\ \{(i, t-1)\} & \text{if } i \text{ is the root and } t > 1 \\ \{(\text{pa}(i), t), (i, t-1)\} & \text{otherwise} \end{cases}\end{aligned}$$

and

$$\text{pa}(i) = \{j | j \text{ is the parent of cell type } i \text{ in the tree}\}$$

Below we define the parameters of our model, which specify the transition probabilities $\mathbb{P}(z_t^i|\text{pa}(z_t^i))$ and emission probabilities $\mathbb{P}(x_t^i|z_t^i)$.

Transition probabilities

The probability of observing state z_t^i conditional on the parent states $\text{pa}(z_t^i)$ is given by

$$\mathbb{P}(z_t^i|\text{pa}(z_t^i)) = \begin{cases} \theta_{mn}^k & \equiv \mathbb{P}(z_t^i = k | z_t^{\text{pa}(i)} = m, z_{t-1}^i = n) & \text{if } t > 1, i \text{ is not root} \\ \alpha_m^k & \equiv \mathbb{P}(z_t^i = k | z_{t-1}^i = m) & \text{if } t > 1, i \text{ is root} \\ \beta_m^k & \equiv \mathbb{P}(z_t^i = k | z_t^{\text{pa}(i)} = m) & \text{if } t = 1, i \text{ is not root} \\ \gamma^k & \equiv \mathbb{P}(z_t^i = k) & \text{if } t = 1, i \text{ is root} \end{cases}$$

The total number of parameters in each transition matrix are: $|\theta| = K^2 \times K$, $|\alpha| = K \times K$, $|\beta| = K \times K$, and $|\gamma| = K$.

Emission probabilities

We treat each histone mark as independent variables, the probability of observing lth histone mark given certain cell state k is given by:

$$e_l^k \equiv \mathbb{P}(x_{t,l}^i = 1 | z_t^i = k) \qquad \forall i = 1 \dots I, t = 1 \dots T$$

where $x_{t,l}^i$ represents the lth histone mark, which takes a binary value of $\{0,1\}$. Thus the emission probability matrix e has $K \times L$ parameters (or K vectors with dimension $1 \times L$).

1.3 Model learning

We use variational EM algorithm to do model learning. The Variational EM algorithm minimizes the free energy

$$F = -\sum_{Z} \mathbb{Q}(Z) \log \frac{\mathbb{P}(X, Z; \Theta)}{\mathbb{Q}(Z)} \tag{1}$$

$$= \mathbb{E}_{\mathbb{Q}(Z)}[\log \mathbb{Q}(Z) - \log \mathbb{P}(X, Z; \Theta)] \tag{2}$$

under some approximate form of hidden variable distribution. The algorithm iterates between two procedures - expectation (or inference) and maximization (or learning). Below we derive the update formula of the E-step and M-step for the mean-field and structured mean-field approximations.

Mean-field approximation

The mean-field approximation assumes the following factorized form of $\mathbb{Q}(Z)$

$$\mathbb{Q}(Z) = \prod_{i} \prod_{t} q(z_t^i) \tag{3}$$

where $q(z_t^i)$ represents the distribution of hidden variable z_t^i of node (i, t).

Under the assumption (3), the first term of (1) becomes

$$\mathbb{E}_{\mathbb{Q}(Z)}[\log \mathbb{Q}(Z)] = \sum_{i} \sum_{t} \sum_{Z} \left(\prod_{i'} \prod_{t'} q(z_{t'}^{i'}) \right) \log q(z_t^i) \tag{4}$$

$$= \sum_{i} \sum_{t} \sum_{z_t^i} q_{it} \log q_{it}.$$

For the last step of (4) and below we abbreviate $q(z_t^i)$ as q_{it} for notational simplicity. The derivation is easily attained by noting that the summation over $\{z_{t'}^{i'}\}$ for all $(i', t') \neq (i, t)$ yields 1. This is an observation that we will use frequently in later derivations.

The second term in the free energy

$$\mathbb{E}_{\mathbb{Q}(Z)}[\log \mathbb{P}(X, Z; \Theta)] = \mathbb{E}_{\mathbb{Q}(Z)} \left[\sum_{i} \sum_{t} (\log \mathbb{P}(z_t^i | \mathrm{pa}(z_t^i)) + \log \mathbb{P}(x_t^i | z_t^i)) \right] \tag{5}$$

$$= \sum_{i} \sum_{t} \sum_{z} \left(\prod_{(i't')} q_{i't'} \right) \left[\log \mathbb{P}(z_t^i | \mathrm{pa}(z_t^i)) + \log \mathbb{P}(x_t^i | z_t^i) \right] \tag{6}$$

$$= \sum_{i} \sum_{t} \sum_{z_t^i, \mathrm{pa}(z_t^i)} \left(q_{it} \prod_{(i't') \in \mathrm{pa}(i,t)} q_{i't'} \right) \left[\log \mathbb{P}(z_t^i | \mathrm{pa}(z_t^i)) + \log \mathbb{P}(x_t^i | z_t^i) \right]$$

$$= \sum_{i} \sum_{t} \left[\sum_{z_t^i, \mathrm{pa}(z_t^i)} \left(q_{it} \prod_{(i't') \in \mathrm{pa}(i,t)} q_{i't'} \right) \log \mathbb{P}(z_t^i | \mathrm{pa}(z_t^i)) + \sum_{z_t^i} q_{it} \log \mathbb{P}(x_t^i | z_t^i) \right]$$

where, assuming there are no missing marks, the emission probability $\mathbb{P}(x_t^i|z_t^i)$ is given by

$$\mathbb{P}(x_t^i|z_t^i = k) = \prod_l \left(I(x_{t,l}^i = 1)e_l^k + (1 - I(x_{t,l}^i = 1))(1 - e_l^k)\right) \tag{7}$$

For the expectation step (E-step), we isolate the terms in (4) and (5) that involve q_{it} :

$$\begin{aligned} F(q_{it}) = & \sum_{z_t^i} q_{it} \log q_{it} - \sum_{z_t^i, \mathrm{pa}(z_t^i)} \left(q_{it} \prod_{(i',t') \in \pi(i,t)} q_{i't'} \right) \left[\log \mathbb{P}(z_t^i|\mathrm{pa}(z_t^i)) + \log \mathbb{P}(x_t^i|z_t^i) \right] \\ & - \sum_{z_{t'}^i, \mathrm{pa}(z_{t'}^i)|t=t'+1} \left(q_{it'} q_{it} q_{\mathrm{pa}(i),t'} \right) \log \mathbb{P}(z_{t'}^i|\mathrm{pa}(z_{t'}^i)) \\ & - \sum_{z_{t'}^{i'}, \mathrm{pa}(z_{t'}^{i'})|(i,t)=(\mathrm{pa}(i'),t')} (q_{i't'} q_{it} q_{i',t'-1}) \log \mathbb{P}(z_{t'}^{i'}|\mathrm{pa}(z_{t'}^{i'})). \end{aligned} \tag{8}$$

The above equation can be written as $\sum_{z_t^i}(q_{it} \log q_{it} - q_{it} \log \phi(z_t^i))$ with

$$\begin{aligned} \log \phi(z_t^i) = & \sum_{\mathrm{pa}(z_t^i)} \left(\prod_{(i',t') \in \mathrm{pa}(i,t)} q_{i't'} \right) \left[\log \mathbb{P}(z_t^i|\mathrm{pa}(z_t^i)) + \log \mathbb{P}(x_t^i|z_t^i) \right] \\ & + \sum_{z_{t'}^{i'}, z_{t'}^{\mathrm{pa}(i')}|t=t'+1} \left(q_{it'} q_{\mathrm{pa}(i),t'} \right) \log \mathbb{P}(z_{t'}^i|\mathrm{pa}(z_{t'}^i)) \\ & + \sum_{z_t^{i'}, z_{t-1}^{i'}|i=\mathrm{pa}(i')} (q_{i't} q_{i',t-1}) \log \mathbb{P}(z_{t'}^{i'}|\mathrm{pa}(z_{t'}^{i'})). \end{aligned} \tag{9}$$

Subsequently q_{it} is obtained by normalizing $\phi(z_t^i)$,

$$q_{it}(z_t^i) = \phi(z_t^i) / \sum_{z_t^i} \phi(z_t^i)$$

The M-step seeks parameters that minimize the free energy F under the constraints $\sum_a \theta_{bc}^a = 1, \sum_a \alpha_b^a = 1, \sum_a \beta_b^a = 1, \sum_a \gamma^a = 1$. where

$$\begin{aligned} F &= \mathbb{E}_Q[-\log \mathbb{P}(X, Z; \Theta)] + C \\ &= -\sum_{i,t} (q_{it} \prod_{(i',t') \in \mathrm{pa}(i,t)} q_{i't'}) \left[\log \mathbb{P}(z_t^i|\mathrm{pa}(z_t^i)) + \log \mathbb{P}(x_t^i|z_t^i) \right] + C, \end{aligned} \tag{10}$$

where the entropy term, denoted as C, is now a constant given fixed $\mathbb{Q}(Z)$. We can group terms associated with each parameter matrix and we can easily see that the parameter values that maximize F are given by the average frequency of occurrence of each child-parent state. More specifically, the parameter updates are

given by

$$\theta_{mn}^{k} \propto \sum_{i>1,t>1} q(z_t^i = k) q(z_t^{\mathrm{pa}(i)} = m) q(z_{t-1}^i = n), \tag{11}$$

$$a_m^k \propto \sum_{i=1,t>1} q(z_t^i = k) q(z_{t-1}^i = m), \tag{12}$$

$$\beta_m^k \propto \sum_{i>1,t=1} q(z_t^i = k) q(z_t^{\mathrm{pa}(i)} = m), \tag{13}$$

$$\gamma^k \propto q(z_1^1 = k), \tag{14}$$

$$e_l^k = \frac{\sum_{i,t} q(z_t^i = k) I(x_{t,l}^i = 1)}{\sum_{i,t} q(z_t^i = k)} \tag{15}$$

where $I(\cdot)$ is the indicator function and $i = 1$ corresponds to the root species.

Structured mean-field approximation

In structured mean-field approximation, we assume that $\mathbb{Q}(Z)$ takes the following form

$$\mathbb{Q}(Z) = \prod_{i=1}^{I} q_i(\mathbf{z}_i),$$

where $\mathbf{z}_i$ is the group of hidden variables within ith chain.

We can write F as

$$\begin{aligned} F &= \mathbb{E}_Q[\log \mathbb{Q}(Z) - \log \mathbb{P}(X, Z; \Theta)] \\ &= \sum_{i=1}^{I} \mathbb{E}_Q[\log q_i(Z_i)] - \sum_{i=1}^{I} \sum_{t=1}^{T} \left(\mathbb{E}_Q[\log \mathbb{P}(z_t^i | \mathrm{pa}(z_t^i))] + \mathbb{E}_Q[\log \mathbb{P}(x_t^i | z_t^i)] \right) \\ &= \sum_{i=1}^{I} \mathbb{E}_{q_i}[\log q_i(\mathbf{z}_i)] - \sum_{i=1}^{I} \sum_{t=1}^{T} \left(\mathbb{E}_{q_i, q_{\mathrm{pa}(i)}}[\log \mathbb{P}(z_t^i | \mathrm{pa}(z_t^i))] + \mathbb{E}_{q_i}[\log \mathbb{P}(x_t^i | z_t^i)] \right). \end{aligned} \tag{16}$$

Again we isolate the terms that involves $q_i(\mathbf{z}_i)$

$$\begin{aligned} F &= \mathbb{E}_{q_i}[\log q_i(\mathbf{z}_i)] - \sum_{t=1}^{T} \left(\mathbb{E}_{q_i, q_{\mathrm{pa}(i)}}[\log \mathbb{P}(z_t^i | \mathrm{pa}(z_t^i))] + \mathbb{E}_{q_i}[\log \mathbb{P}(x_t^i | z_t^i)] \right) \\ &\quad - \sum_{i' | i \in \mathrm{pa}(i')} \sum_{t=1}^{T} \mathbb{E}_{q_{i'}, q_i}[\log \mathbb{P}(z_{t'}^{i'} | \mathrm{pa}(z_{t'}^{i'}))] + const \\ &= \mathbb{E}_{q_i}[\log q_i(\mathbf{z}_i))] - \sum_{t=1}^{T} \mathbb{E}_{q_i} \left[\log f_{it}(z_t^i, z_{t-1}^i) + \log \mathbb{P}(x_t^i | z_t^i) \right], \end{aligned} \tag{17}$$

where we have defined

$$f_{it}(z_t^i, z_{t-1}^i) \equiv \exp \left(\mathbb{E}_{q_{\mathrm{pa}(i)}}[\log \mathbb{P}(z_t^i | \mathrm{pa}(z_t^i))] + \sum_{i' | i \in \mathrm{pa}(i')} \mathbb{E}_{q_{i'}}[\log \mathbb{P}(z_t^{i'} | \mathrm{pa}(z_t^{i'}))] \right).$$

Equation (17) from above shares the form of the free energy of a hidden Markov model if $f_{it}(z_t^i, z_{t-1}^i)$ has the normalization property of a transition matrix. To normalize it, we use the following procedure, starting from the last node in the chain

Initialize: $g(z_T^i) = [1, \ldots, 1]$

For $t = T : 2$

Calculate $\tilde{f}(z_t^i, z_{t-1}^i) = g(z_t^i) f(z_t^i, z_{t-1}^i)));$

$g(z_{t-1}^i) = \sum_{z_t^i} \tilde{f}(z_t^i, z_{t-1}^i);$

$\tilde{f}(z_t^i, z_{t-1}^i) = \tilde{f}(z_t^i, z_{t-1}^i)/g(z_{t-1}^i).$

For $t = 1$, we get $\log(f_{i1}(z_1^i)g(z_1^i))$, which we can normalize to get the prior distribution of variable z_1^i.

$$\tilde{f}(z_1^i) = \frac{f_{i1}(z_1^i)g(z_1^i)}{\sum_{z_1^i} f_{i1}(z_1^i)g(z_1^i)}$$

where $f_{i1}(z_1^i) = \exp\left(\mathbb{E}_{q_{\text{pa}(i)}}[\log \mathbb{P}(z_1^i | z_1^{\text{pa}(i)})] + \sum_{i' | i \in \text{pa}(i')} \mathbb{E}_{q_{i'}}[\log \mathbb{P}(z_1^{i'} | z_1^i)]\right)$.

Notice that now $\tilde{f}$ is properly normalized. Next we can use the forward-backward algorithm to infer the posterior distribution $q_i(z^i)$, thus the marginal $q(z_t^i)$ and $q(z_t^i, z_{t-1}^i)$. Omitting index i and defining the forward and backward messages $a_t^k = \mathbb{P}(x_1, \ldots, x_t, z_t = k)$ and $b_t^k = \mathbb{P}(x_{t+1}, \ldots, x_T | z_t = k)$, we can then perform forward-backward algorithm as below:

Forward algorithm

Initialization: $a_1^k = e_k(x_1)\tilde{f}(z_1 = k);$

For $t = 2 : T$

$$a_t^k = e_k(x_t) \sum_j a_{t-1}^j \tilde{f}(z_{t-1} = j, z_t = k).$$

where $\tilde{f}(z_1 = k), \tilde{f}(z_{t-1} = j, z_t = k)$ are the normalized prior and transition matrices calculated previously. $e_k(x_t) = \mathbb{P}(x_t | z_t = k) = \prod_{l=1}^{L}[(1 - e_l^k)I(x_{t,l} = 0) + e_l^k I(x_{t,l} = 1)]$.

Backward algorithm

Initialization: $b_T^k = (1, 1, \ldots, 1);$

For $t = T-1 : 1$:

$$b_t^k = \sum_j \tilde{f}(z_t = k, z_{t+1} = j) e_l(x_{t+1}) b_{t+1}^j.$$

The likelihood is given by $\mathbb{P}(x) = \sum_{z_T} \mathbb{P}(x, z_T) = \sum_k a_T^k$. We can calculate the posterior distribution of hidden variables $\mathbb{P}(z_t = k|x)$ and $\mathbb{P}(z_t = k, z_{t+1} = j|x)$ as

$$q(z_t = k|x) = \frac{a_t(z_t = k) b_t(z_t = k)}{\mathbb{P}(x)},$$
$$q(z_t = k, z_{t+1} = j|x) = \frac{a_t(z_t = k)\tilde{f}(z_t = k, z_{t+1} = j) e_l(x_{t+1}) b_t(z_{t+1} = j)}{\mathbb{P}(x)}.$$

The M-step is similar to the mean-field case and is given below.

$$\theta_{mn}^k \propto \sum_{i>1,t>1} q(z_t^{\mathrm{pa}(i)} = m) q(z_{t-1}^i = n, z_t^i = k),$$
$$\alpha_m^k \propto \sum_{i=1,t>1} q(z_{t-1}^i = m, z_t^i = k),$$
$$\beta_m^k \propto \sum_{i>1,t=1} q(z_t^{\mathrm{pa}(i)} = m) q(z_t^i = k),$$
$$\gamma^k \propto q(z_1^1 = k),$$
$$e_l^k = \frac{\sum_{i,t} q(z_t^i = k) I(x_{t,l}^i = 1)}{\sum_{i,t} q(z_t^i = k)}$$

1.4 Incorporating missing markers and hidden cell types

Many additional histone modifications are available beyond the nine included in the ENCODE dataset. Most of the additional marks are only available for a small number of cell types. These markers, though excluded from the current analysis, could be incorporated to provide additional model refinement. Since each mark is treated as an independent variable, if a certain marker l is absent in some of the cell types, we simply remove the lth emission term in (7). While the inference step and parameter learning of the transition matrices follow the exact procedure, the estimation of emission parameters in the M-step is modified as

$$e_l^k \propto \sum_{i,t} q(z_t^i = k) I(x_{t,l}^i = 1) I(M_l^i = 1),$$

where I is the indicator function and M is a binary matrix with M_l^i indicating the availability of lth marker .

Next we discuss the incorporation of hidden cell types. In the current approach we use simple, biologically-motivated tree structures for the human dataset. In reality, cells differentiate through multiple steps, and some of the intermediate cell types may not have available experimentally derived measurements. Also,

other statistically motivated tree structures such as those created by hierarchical clustering could be used. Adding unobserved cell types has both biological and modeling significance. Computationally, hidden cell types can be treated as a special case of absent markers by considering all of its markers as missing.The inference step and parameter learning are similar to the above case of absent markers.

1.5 Artificial data

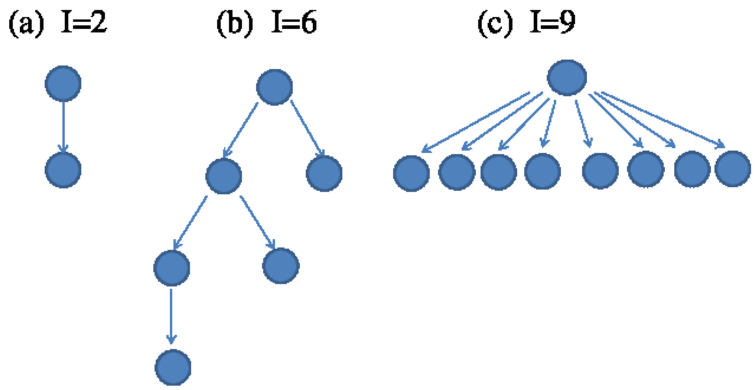

Figure 1: **Lineage trees used for artificial data**

We assess the accuracy of different approximations on artificial datasets generated under the TreeHMM model described above. We tested cases with different tree structures ($I \in \{2, 6, 9\}$) and number of states $K \in \{5, 10\}$ .For the cases of $I = 2$, we consider a lineage where species are connected in a line structure. For the case of $I = 9$, we adopt a tree structure with one being root and others being direct children. And for the case of $I = 6$, we have used a relatively deep tree, as depicted in Fig. 1.5. We also set the number of markers to be $L = 10$ respectively to mimic the real data. Parameters in α, β, γ and e are generated randomly between $[0, 1]$ and normalized to be a conditional probability table (except e). Sub-matrices $\theta(k, :, :)$ are generated by adding perturbations to α. We specify the model in the Bayes Net Toolbox (BNT) [2] and use the *sample_bnet* function in BNT to generate values for the observed nodes using the "true" parameter values. We generate datasets consisting of $T = 10^4$ slices for $K = 5$ and $T = 10^5$ for $K = 10$.

We run each variational EM algorithms on the generated artificial datasets. Since EM algorithm could reach local optimal solutions, we run each algorithm 5 times with random initializations for each dataset and take the result with the lowest free energy. We use the root mean squared error (RMSE) of the elements in each parameter matrix to measure the accuracies of the inferred parameters. To test the consistency of the

learning algorithms, we generated five datasets using different parameters for each (K, I) pair, and record the averaged result of RMSE of inferred parameters w.r.t. the true ones in each parameter matrix.

From results summarized in Table 1, we observe that the SMF approximation consistently outperforms LBP and MF. Although LBP and MF can give good estimates of the emission matrix e for some simple cases (e.g. $K = 5$), they usually give a large error in the learned α and θ parameters, indicating that the two methods are less accurate in inferring joint marginals of hidden variables. We also observe that the performance of LBP decreases with increasing number of leaf cell types (e.g., percent error of $\alpha = 4.5\%$ for $I = 2$ versus 26% for $I = 9$ in $K = 5$ cases), indicating that LBP is sensitive to the tree structures and tends to infer inaccurate marginals when nodes in the graph have many connected nodes. Interestingly it performs well in I=6 case of relatively deep lineage tree. In contrast, SMF consistently gives accurate estimations for all the cases. SMF's advantage is especially apparent for estimating α and θ, which involve the joint marginal. For example, the percent error of $\theta = 9.4\%$ versus 63% for MF and 168% for LBP in the $(I, K) = (9, 10)$ case. Also we can see that, as expected, more data is needed to achieve the same accuracy for the inferred parameters as the number of states K (and therefore the number of parameters) increases.

Table 1: Accuracy of different algorithms in recovering parameter values using artificial data.

		RMSE (percent*)		
(K, I, T)	Approx	e	α	θ
(5, 2, 10K)	SMF	0.008 (1.5%)	0.009 (4.6%)	0.022 (11%)
	MF	0.03 (6.7%)	0.03 (17.1%)	0.04 (22%)
	LBP	0.011 (2.1%)	0.009 (4.5%)	0.28 (140%)
(10, 2, 100K)	SMF	0.005 (1.0%)	0.005 (4.8%)	0.013 (13%)
	MF	0.08 (15%)	0.055 (55%)	0.06 (59%)
	LBP	0.06 (12%)	0.026 (26%)	0.17 (170%)
(5, 9, 10K)	SMF	0.004 (0.8%)	0.009 (4.7%)	0.008 (4.2%)
	MF	0.04 (0.9%)	0.03 (18%)	0.04 (19%)
	LBP	0.022 (4.4%)	0.10 (52%)	0.30 (150%)
(10, 9, 100K)	SMF	0.009 (1.8%)	0.008 (8.3%)	0.009 (9.4%)
	MF	0.06 (23%)	0.05 (65%)	0.05 (63%)
	LBP	0.14 (26%)	0.20 (196%)	0.17 (168%)
(5, 6, 10K)	SMF	0.005 (1.0%)	0.015 (7.5%)	0.021 (11%)
	MF	0.04 (8.0%)	0.16 (80%)	0.16 (80%)
	LBP	0.04 (8.0%)	0.014 (70%)	0.08 (40%)

SMF: structured mean field, MF: mean field, LBP: loopy belief propagation
*Each value inside the bracket is the percent error relative to the mean value of the corresponding parameter matrix elements.

1.6 Model complexity for human ENCODE dataset

We seek to determine the model complexity, i.e. the number of chromatin states K, that is best supported by the human histone data. We expanded SMF inference to include all of chromosome 22 and all nine cell types, and varied the number of hidden states. Supplemental Figure 2 shows the free energy together with the complexity-penalized score according to Bayesian information criterion (BIC), assuming SMF free

energy to be a close approximation to the true likelihood. To proceed with whole-genome analysis, we chose $K = 18$ where the maximum BIC score is achieved in this smaller dataset. We note that the value is close to the number of states (15) selected by [1] which was chosen partly by post-analysis of the assigned states.

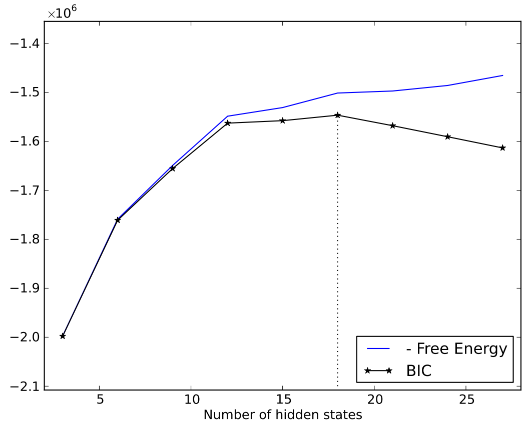

Figure 2: **Choosing model complexity (the optimal number of states K).** We ran the SMF variational EM algorithm on chromosome 22 with a range of K values . The final free energy for different K and the complexity-penalized likelihood score (BIC) is shown, revealing that a model with between 15 and 20 states are well-supported by the data.

2 Supplementary tables and figures

State	mean	H1 ES	K562	GM12878	HePG2	HUVEC	HSMM	NHLF	NHEK	HMEC
1	1.0	2.02	0.72	0.95	0.17	0.87	0.94	0.84	1.21	1.28
2	0.7	0.39	0.59	1.15	2.84	0.42	1.1	1.14	0.68	0.7
3	1.1	0.58	0.97	1.17	2.04	0.58	0.88	1.04	0.91	0.83
4	0.1	0.84	2.21	0.85	0.87	0.9	0.82	0.82	0.84	0.85
5	1.5	0.4	0.53	0.78	0.05	1.54	1.46	1.15	1.43	1.66
6	4.0	1.08	1.05	0.88	1.36	0.81	0.79	0.89	0.94	1.19
7	4.3	0.24	1.01	1.2	1.12	0.89	1.67	0.98	0.98	0.91
8	0.7	1.26	0.56	1.02	1.85	0.7	0.99	0.97	0.81	0.84
9	1.3	2.93	0.99	0.88	0.61	0.91	0.91	0.61	0.51	0.66
10	0.5	0.09	0.48	0.8	3.54	0.36	1.06	1.21	0.73	0.74
11	1.1	0.18	1.57	1.46	0.15	1.56	1.0	0.68	1.48	0.93
12	11	5.29	0.42	0.38	0.47	0.37	0.45	0.53	0.48	0.61
13	0.8	0.12	1.61	1.14	0.9	0.86	1.7	0.95	1.03	0.69
14	1.3	1.04	1.19	0.95	0.95	0.96	0.96	1.14	1.04	0.76
15	1.6	2.09	1.26	0.36	1.65	1.06	0.78	0.41	0.9	0.48
16	15	0.76	0.95	0.98	0.99	0.96	1.19	1.07	1.11	1.0
17	6.5	0.35	1.43	0.46	1.82	1.43	0.88	1.01	1.08	0.54
18	48	0.21	1.08	1.23	0.95	1.13	1.03	1.13	1.08	1.17
	(%)				(fold)					

Table 2: **Average coverage and cell type-specific enrichment of learned chromatin states.** The "mean" column gives the average percent coverage, or the fraction of the segmented genome which is covered by the state. The following columns show the enrichment of each state in each cell type relative to the across-species mean (column 2).

Precision/Recall	H1-ES	K562	K562(polyA+)
TreeHMM	0.441/0.577	0.344/0.536	0.324/0.635
ChromHMM	0.353/0.592	0.338/0.530	0.319/0.630

Table 3: **Precision(first number) and Recall(second number) of inferred promoter states for TreeHMM and ChromHMM using CAGE-determined TSS in H1-ES and K562.** TSS's are defined as regions with at least two CAGE tags within ± 500 bp. H1-ES: whole-cell RNA, K562: cytoplasmic RNA, K562(polyA+): poly-A enriched cytoplasmic RNA.

References

1. Ernst J, Kheradpour P, Mikkelsen TS, Shoresh N, Ward LD, Epstein CB, Zhang X, Wang L, Issner R, Coyne M, Ku M, Durham T, Kellis M, Bernstein BE: **Mapping and analysis of chromatin state dynamics in nine human cell types.** *Nature* 2011, **473**(7345):43–9.
2. Murphy K, et al.: **The Bayes net toolbox for matlab**. *Computing science and statistics* 2001, **33**(2):1024–1034.

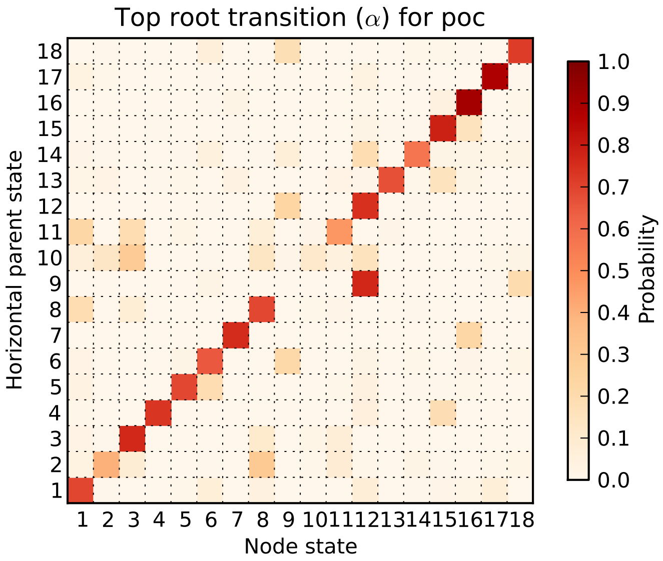

Figure 3: **Learned transition matrix α for the root cell type (H1 ES).**

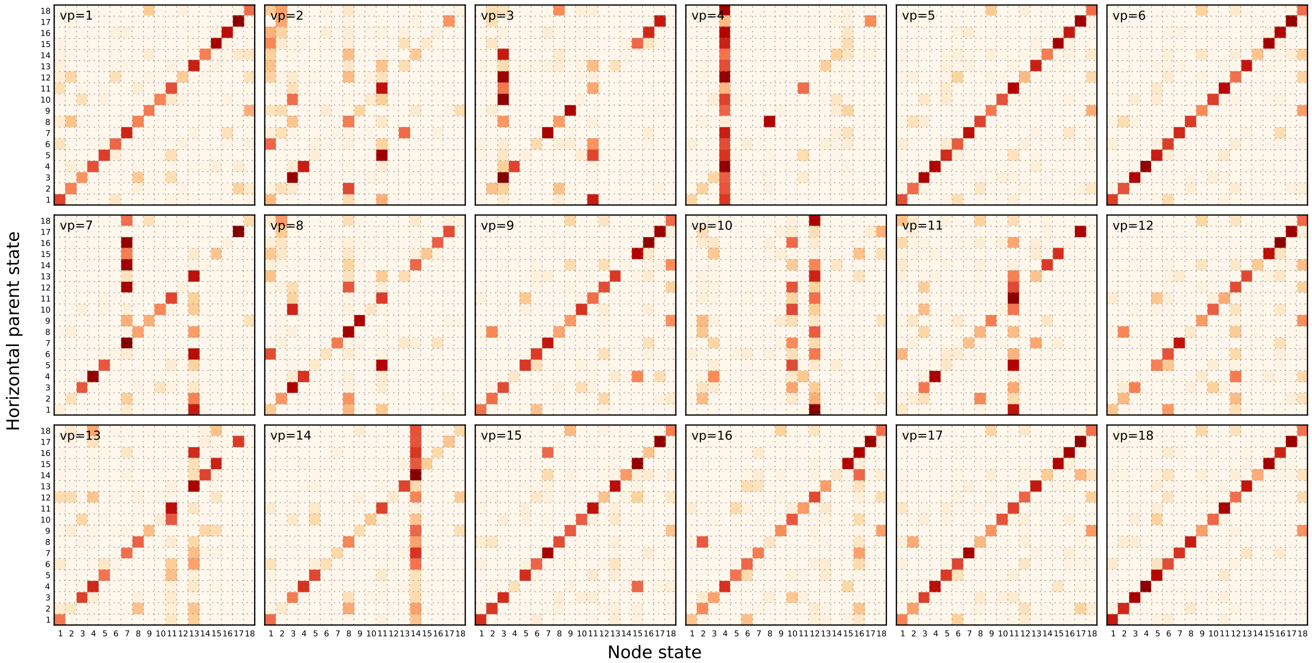

Figure 4: **Transition matrix θ shown as specific submatrices for each vertical parent state** Each sub-matrix is associated with vertical parent state 1 to 18 indicated at the top-left. A strong diagonal in the transition matrix indicates spatial persistence of states, while a strong vertical line indicates persistance from the corresponding vertical state across cell types.

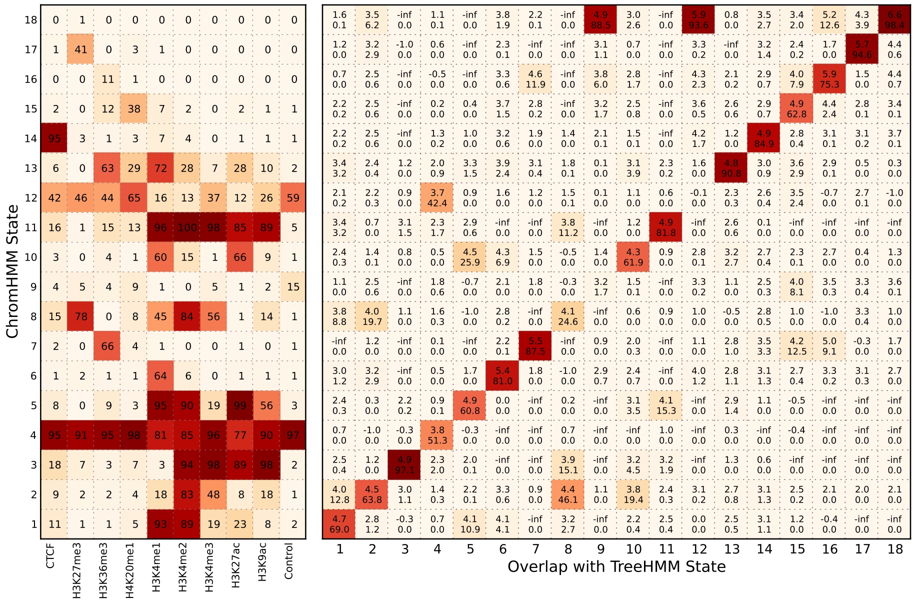

Figure 5: **Comparison with ChromHMM.** Left panel: Emission probabilities learned by ChromHMM with $K = 18$. Right panel: Confusion matrix showing the total number of bases identified as belonging to a particular state by the two methods. In each cell, the top number is the log_{10}(number of bins) in the intersection, the value below corresponds to the percentage in the corresponding tree-HMM sites and is used to color the heatmap. (values in each column sum up to 1.)

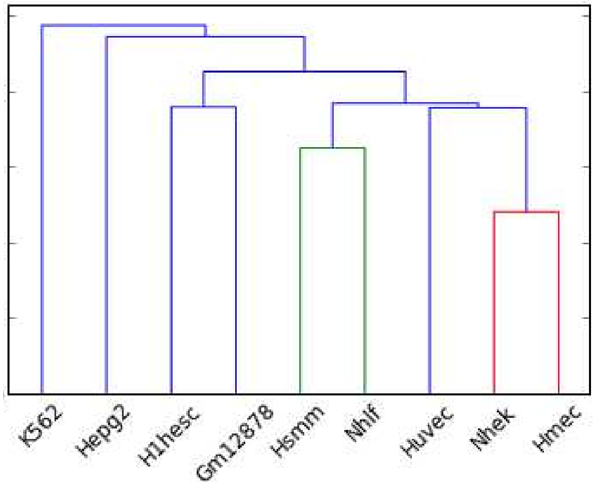

Figure 6: Hierarchical clustering of binarized histone marks in human. Branch lengths indicate the manhattan distance between species, considering all histone marks. The two most similar cell types are indicated in red, and the next two most similar are indicated in green.

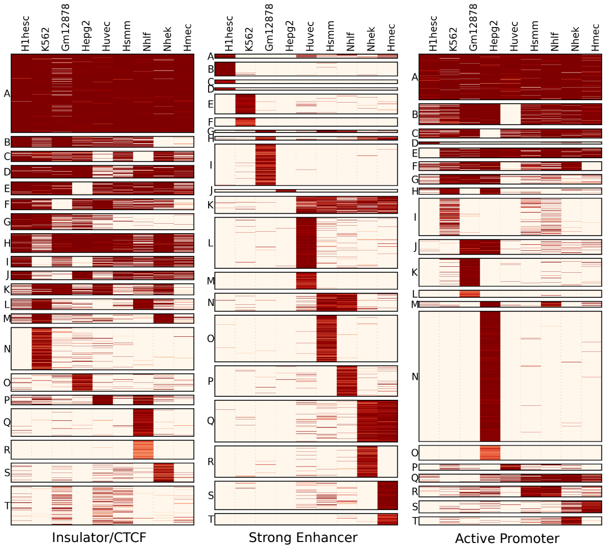

Figure 7: **K-means clustering reveals different cell-type specificities for different chromatin states.** K-means clustering with 20 clusters was performed on the posterior probability of all the bins being in each state, posterior probability is shown as heatmap color, cell types are indicated at the top of each column. From left to right: Insulator/CTCF (state 14), Strong Enhancer (state 5), and Active Promoter (state 3).
